# Clinical outcomes and toxicity after proton- versus photon-based spatially fractionated radiotherapy for soft-tissue sarcoma: a retrospective cohort

**DOI:** 10.3389/fonc.2026.1741156

**Published:** 2026-10-28

**Authors:** Adeniyi A. Olabumuyi, Matthew H. Brown, Soha Bazyar, Sina Mossahebi, James W. Snider, Dan Kunaprayoon, Vincent Y. Ng, William F. Regine, Jason K. Molitoris

**Affiliations:** 1 Department of Radiation Oncology, University of Maryland Medical Center, Baltimore, MD, United States; 2 LifeBridge Health, Baltimore, MD, United States; 3 University of Maryland School of Medicine, Baltimore, MD, United States; 4 Partners in Healthcare Technology, LLC, Alpharetta, GA, United States

**Keywords:** cancer, GRID, lattice, radiotherapy, sarcoma, SFRT

## Abstract

**Introduction:**

Spatially fractionated radiotherapy (SFRT) enables focal dose escalation in bulky/high-grade STS. Proton SFRT, leveraging its Bragg peak, reduces low-dose exposure but may increase proximal skin dose, potentially influencing perioperative wound complications. Comparative data remain limited.

**Methods:**

We retrospectively reviewed 37 high-grade or bulky STS patients (2009–2023) treated with preoperative SFRT (photon or proton GRID/Lattice) followed by conventional fractionated radiotherapy (CFRT) and surgery. Toxicities (including major wound complications per NCIC criteria) and oncologic outcomes were analyzed using Fisher’s exact tests and Kaplan–Meier.

**Results:**

Median follow-up was 40 months. Sixteen patients (43.2%) received proton SFRT; all received CFRT (median: 50.4 Gy). Major wound complications 6/16 (37.5%) proton SFRT vs 8/21 (38.1%) photon SFRT patients, with no significant difference (P = 1.000). Acute grade 2-3 (6/16 vs 11/21) and late (3/16 vs 9/21) toxicities did not differ significantly between modalities; no grade 4 or 5 toxicities occurred. Major pathological response (MPR, ≥80% necrosis) was achieved in 12/35 evaluable patients (34.3%) and was not associated with SFRT type (P = 0.489). In exploratory analysis, MPR was associated with longer disease-free survival (median 80 vs. 27 months, P = 0.014). Proton SFRT plans had significantly higher dose heterogeneity (D90/D10 = 0.038 vs 0.093, P = 0.04).

**Discussion:**

In this largest-to-date retrospective cohort of neoadjuvant proton- versus photon-based SFRT + CFRT for STS, no statistically significant differences in perioperative or skin toxicity were observed, and MPR (≥80% necrosis) occurred in 34.3% of evaluable patients; given the small, non-randomized sample these results should be considered hypothesis-generating.

## 1 Introduction

Soft-tissue sarcomas (STS) are a heterogeneous group of malignancies arising from mesenchymal tissues. Although considered rare, data from the SEER database reveals an increasing incidence, from 3.0 per 100,000 persons in 2000 to 3.5 per 100,000 in 2021 (1). The American Cancer Society estimates 13,520 new cases of STS and 5,420 deaths in 2025 in the United States (2).

Surgery remains the cornerstone of curative treatment, particularly for extremity STS. However, high-grade bulky tumors treated with surgery alone often result in suboptimal outcomes. These challenges are mitigated by the addition of neoadjuvant or adjuvant radiation therapy, which significantly improves local disease control (3–5). There is evidence from the Sarcoma Meta-analysis Collaboration (SMAC meta-analysis) to show that adjuvant chemotherapy marginally improves the local recurrence, distant recurrences, overall recurrence in localized resectable STS (6). A recent publication by Mowery et al. demonstrated that neoadjuvant and adjuvant pembrolizumab in addition to surgery and radiation treatment improves disease free survival in stage III undifferentiated pleomorphic sarcoma and pleomorphic/dedifferentiated liposarcoma (7).

Historically, soft-tissue sarcomas have been considered relatively radioresistant (8, 9). Dose escalation has been explored as a strategy to overcome this limitation but is often accompanied by increased toxicity to surrounding organs at risk (OAR). Low α/β of sarcomas suggest potential responsiveness to hypofractionation. This has led to exploration of techniques such as stereotactic body radiation therapy (SBRT), which delivers high doses per fraction and has been proven to be successful in other traditionally radioresistant malignancies like renal cell carcinoma. However, the large size and irregular anatomy of many STS lesions often limit the feasibility of SBRT.

Spatially fractionated radiation therapy (SFRT), introduced in the 1950s with GRID technology, offers a promising strategy to overcome radioresistance by delivering high doses to discreet subvolumes of the tumor while minimizing toxicity (10). SFRT also may change the tumor microenvironment through immunogenic, bystander, and microvasculature effects, complementing a standard fractionated radiotherapy approach (11, 12). This technique creates a dosimetric pattern of peaks and valleys, with peaks receiving ablative doses and valleys receiving lower doses (13). Initially implemented using physical GRID blocks, SFRT has since evolved with the advent of multi-leaf collimators (MLC), lattice techniques and proton pencil beam scanning, enabling delivery with both photons and protons (14). A study by Neuner et al. comparing MLC to Cerrobend revealed no difference in pain response and local control (15). Lattice RT involves a radiating technique that delivers 3D spheres with high dose regions (hotspots) within the GTV known as vertices surrounding by low-dose cold areas (13). These methods can be particularly advantageous in bulky high-grade soft tissue sarcomas given their radioresistant nature, substantial tumor burden during treatment, and the observed suboptimal local control rates.

In comparison to photon SFRT, proton SFRT are proven to have higher heterogeneity expressed as D90/D10 (16, 17). Due to the Bragg’s peak effect, they are also able to have no dose delivery beyond the target (16, 17). Studies also note that the low-dose bath is reduced with proton SFRT, with lower volumes of the normal tissue that received 5% of the prescribed dose (V5%) (14). They are thus advantageous in treating deep seated tumors, for re-irradiation and for treating pediatric patients (14, 17). However, doses to the surface and tissue proximal to the target in the beam path are higher with proton SFRT compared to photon SFRT (17, 18). This indicates there could be higher skin toxicities/wound complications with proton SFRT compared to photons, however there is limited literature comparing the modalities. An early results study by Mohiuddin et al. on 10 patients (50% sarcomas) all treated with proton therapy indicated that 50% of the patients had Grade 1 and 2 skin toxicities with no Grade 3 toxicities with a median follow up of 5.9 months (19).

The O’Sullivan et al. study demonstrated the benefits of preoperative radiation therapy in reducing treatment volume compared to postoperative approaches (20). However, pCR remains relatively rare amongst bulky or high grade STS. This is demonstrated in previous clinical trials. RTOG 0630 which trialed a reduced target volume had a pCR of 19.4%. While RTOG 9514 in which patients received sequential chemoradiation had a pCR of 27.5% (21). Our institution developed a multidisciplinary protocol for managing bulky STS. This approach integrates neoadjuvant SFRT (12–15 Gy in one fraction), followed by conventionally fractionated radiation therapy and oncologic surgical resection for high grade and/or bulky STS. In our institution we use major pathological response (defined as ≥80% necrosis) as a surrogate for pCR. Our hypothesis was this treatment strategy would lead to favorable major pathological response (MPR) rate and toxicity outcomes; consistent with our previously published initial report (22). We, thus decided to evaluate the difference in skin toxicities, particularly perioperative/post-operative wound complications in our cohort of patients.

## 2 Methodology

### 2.1 Patients

This retrospective, single-institution, IRB approved (approval number HP-00084301), study was conducted at a tertiary medical center and affiliated proton center. Patients diagnosed with sarcoma and treated with SFRT between January 1, 2009, and December 31, 2023, were retrospectively reviewed under an Institutional Review Board approved protocol. Included patients had biopsy confirmed, non-metastatic, STS that was grade 2–3 and/or sized greater than 6.5cm in maximal dimension. All evaluated patients underwent a single SFRT treatment followed by conventionally fractionated radiotherapy (CFRT) and surgical resection. Patients were excluded from analysis if they received SFRT combined with palliative local treatment, if they did not receive neoadjuvant CFRT, or if they did not undergo definitive surgical resection following completion of CFRT.

### 2.2 Radiation planning and delivery

In both photon and proton GRID treatment methods, the gantry angle is selected with consideration of surrounding OARs to minimize unintended dose exposure. All treatment planning is performed in RayStation (RaySearch Laboratories; Stockholm, Sweden). For photon GRID, beamlets are automatically placed using an in-house script that arranges the MLCs into a GRID pattern. Beam energy is selected based on tumor depth. Monitor units are determined using institutionally commissioned GRID output factors derived from dosimetric measurements for the specific GRID configuration and field size. The resulting treatment plan is subsequently calculated, reviewed, and verified within the treatment planning system prior to clinical delivery. Photon lattice plans are generated using 1-cm-diameter spherical structures (vertices) spaced 3–3.5 cm apart within the GTV, created using an in-house script within the treatment planning system. A photon-VMAT lattice plan is then developed using 6 MV photon beams with 5–8 arcs.

In proton GRID, virtual cylindrical targets (3 mm in diameter, spaced 2–3 cm apart) are placed within a GTV contracted by 5–10 mm to account for beam end-ranging and setup uncertainties, helping to spare normal tissue. Proton spots are optimized to deliver a homogeneous high dose to each cylinder while maintaining low-dose regions between them, achieving a high peak-to-valley dose ratio (PVDR). For proton lattice therapy, a robust proton lattice (RPL) method is used (please see ref 22 for details of our proton lattice technique), incorporating a primary beam (PB) and a robust complementary beam (RCB) (23). The PB delivers approximately 67% of the dose (8 Gy) to the vertices, while the RCB contributes the remaining 33% (4 Gy) and is robustly optimized against ±3–5 mm setup and ±3.5–5% range uncertainties.

Following SFRT, patients underwent conventionally fractionated external beam radiation therapy (EBRT). The prescription dose and additional treatment parameters were determined at the discretion of the treating radiation oncologist. Treatment modality was nonrandomized and selected by the multidisciplinary team/physician discretion according to tumor anatomy, technical feasibility, anticipated dose distribution, proton availability/insurance approval, and patient-specific logistical considerations. Surgical resection was performed 5–7 weeks after the completion of CFRT.

### 2.3 Statistical methods

Data retrieved from medical records included demographic details, Eastern Cooperative Oncology Group (ECOG) performance status, and tumor characteristics such as location, radiologic size, histologic type and grade. Treatment outcomes, including surgical margin status (positive margin defined as tumor at the margin), and post-treatment pathologic parameters e.g., major pathological response [MPR], defined as ≥80% necrosis per our institutional policy as previously published (22), were also recorded.

Dosimetric parameters and treatment outcomes, such as local recurrence and disease progression, were summarized using descriptive statistics, including frequency tables and charts. The relationship between tumor size and treatment effect (percent necrosis) was visualized with scatter plots. Crosstabulations analyzed the association between MPR and SFRT modality (photon vs. proton) using Fisher’s exact test. The association between margin status and local recurrence was also analyzed with a crosstabulation.

Disease-free survival (DFS) was evaluated using Kaplan-Meier survival analysis, and comparisons were made using the log-rank test. The association between major wound complications, defined per NCIC trial, and SFRT modality (photon vs. proton) was analyzed with a crosstabulation and Fisher’s exact test. Other radiation toxicities were reported using frequency tables.

Ninety-five percent confidence intervals (95% CI) for proportions of MPR and major wound complication were done with Wilson score method. While the 95% CI for the proton vs photon sub-group differences in proportions of major wound complication, acute grade 2 and 3 toxicities, and late toxicities were done with Newcombe continuity corrected method. The 95% CI for the proton vs photon sub-group risk ratios of major wound complication, acute grade 2 and 3 toxicities and, late toxicities were done with the Miettinen–Nurminen score method (Koopman method).

## 3 Results

### 3.1 Patient, cancer and treatment characteristics

Thirty-seven consecutive patients (median age of 59, range [18–87 years] were treated from 8/2009-12/2023 met inclusion criteria for analysis (**Table 1**) and the majority, 22 (59.5%), were undifferentiated pleomorphic sarcoma/unclassified pleomorphic sarcoma. The most prevalent histologic grade was grade 3 (G3) in 21 (56.8%) cases. The median largest (pre-treatment, radiologic) tumor dimension was 11.8cm (range: 6.5-30cm) and 16 tumors (43.3%) were ypT3. ypT4 disease was more frequent in the proton group (6 of 16, 37.5%) than in the photon group (1 of 21, 4.8%; p = 0.054) (**Supplementary Table S1**). Extremity sarcomas were the most common tumor site, comprising 28 patients (75.7%). There was no significant difference in the characteristics of the patients/tumors who received photon SFRT compared to proton SFRT.

**Table 1:** Demographic and clinicopathologic data.

Variable	Categories	n (%)
Gender		
	Male	22 (59.5)
	Female	15 (40.5)
Race		
	White	21 (56.8)
	Black	12 (32.4)
	Asian	1 (2.7)
	Other	3 (8.1)
ECOG PS		
	0	27 (73.0)
	1	8 (21.6)
	2	2 (5.4)
SFRT		
	Photon	21 (56.8)
	Proton	16 (43.2)
Histological Grade		
	G1	1 (2.7)
	G2	11 (29.7)
	G3	21 (56.8)
	Ungraded	4 (10.8)
Median size (cm)	11.8 (6.5-30)	
T Stage*		
	ypT1	1 (2.7)
	ypT2	13 (35.1)
	ypT3	16 (43.3)
	ypT4	7 (18.9)
Site		
	Extremity	28 (75.7)
	Non-extremity	9 (24.3)
Major Pathologic Response**		
	No	23 (65.7)
	Yes	12 (34.3)
	Unreported	2
Local recurrence post treatment		
	No	30 (81.1)
	Yes	7 (18.9)

* ypT is a post treatment pathologic variable not a baseline characteristic.

** Percentages are on 35 evaluable patients.

Following SFRT, patients received CFRT with total doses ranging from 45 Gy to 61.33 Gy. Twenty-nine (78%) patients received a total dose of 50.4 Gy. Nine patients received hyperthermia (as deep thermal therapy or external thermal therapy). Thirteen patients received neoadjuvant systemic therapy. Of these, two patients received AIM chemotherapy, ten received Durvalumab and Tremelimumab on the NEXIS clinical trial (24) and one received Pembrolizumab.

Proton SFRT vs Photon SFRT dosimetric comparisons were done in 34 of 37 patients whose plans werereviewable (**Supplementary Figure S1**). A Mann-Whitney U test revealed the proton GRID plans had a significantly lower D90/D10 (median = 0.038, IQR 0.014-0.084) compared to photon GRID plans (median = 0.093, IQR 0.068-0.164) P = 0.04. However, the Mann-Whitney U test comparison of the volume of GTV receiving 95% of the prescribed dose (V95%) revealed that proton GRID plans were higher (median = 1.94%, IQR 1.69%-2.30%), compared to photon GRID plans (median = 0.24%, IQR 0.06%-0.87%) P = <0.001.

### 3.2 Clinical outcomes

The median follow-up period was 40 months (range 5-168months). Local recurrences were observed in 7 patients (18.9%; 95% CI 8% - 35%) (**Table 1**), with an overall median freedom from local recurrence of 80 months (**Supplementary Figure S2**>). Disease progression, defined as either local recurrence or distant metastasis, occurred in 17 patients (45.9%). Negative surgical margins were achieved in 32 patients (86.5%). Major pathological response (MPR) was achieved in 12 of 35 evaluable patients (34.3% (CI: 20.8%-50.8%)); MPR was unavailable in 2 patients. There was a significant association between margin status and local recurrence, with 80% of patients with positive margins developing local recurrences (P = 0.002) (**Supplementary Table S2**). A scatter plot examining the relationship between tumor size and pathological necrosis revealed no significant correlation between the largest pre-treatment tumor dimension and treatment response (**Supplementary Figure S3**).

In exploratory Kaplan-Meier survival analysis, patients with MPR had significantly longer disease-free survival (DFS) compared to those without MPR (median DFS of 80 months vs. 27 months; log-rank P = 0.014) (**Figure 1a**). However, no significant association was found between MPR and overall survival (OS) (**Figure 1b**). Only one patient received proton lattice. Analysis of the relationship between radiation modality (proton vs. photon) vs MPR and local recurrence (LR) using a 2×2 contingency table showed no statistically significant association (P = 0.489, P = 1.000 respectively) (**Supplementary Table S3**).

**Figure 1:**
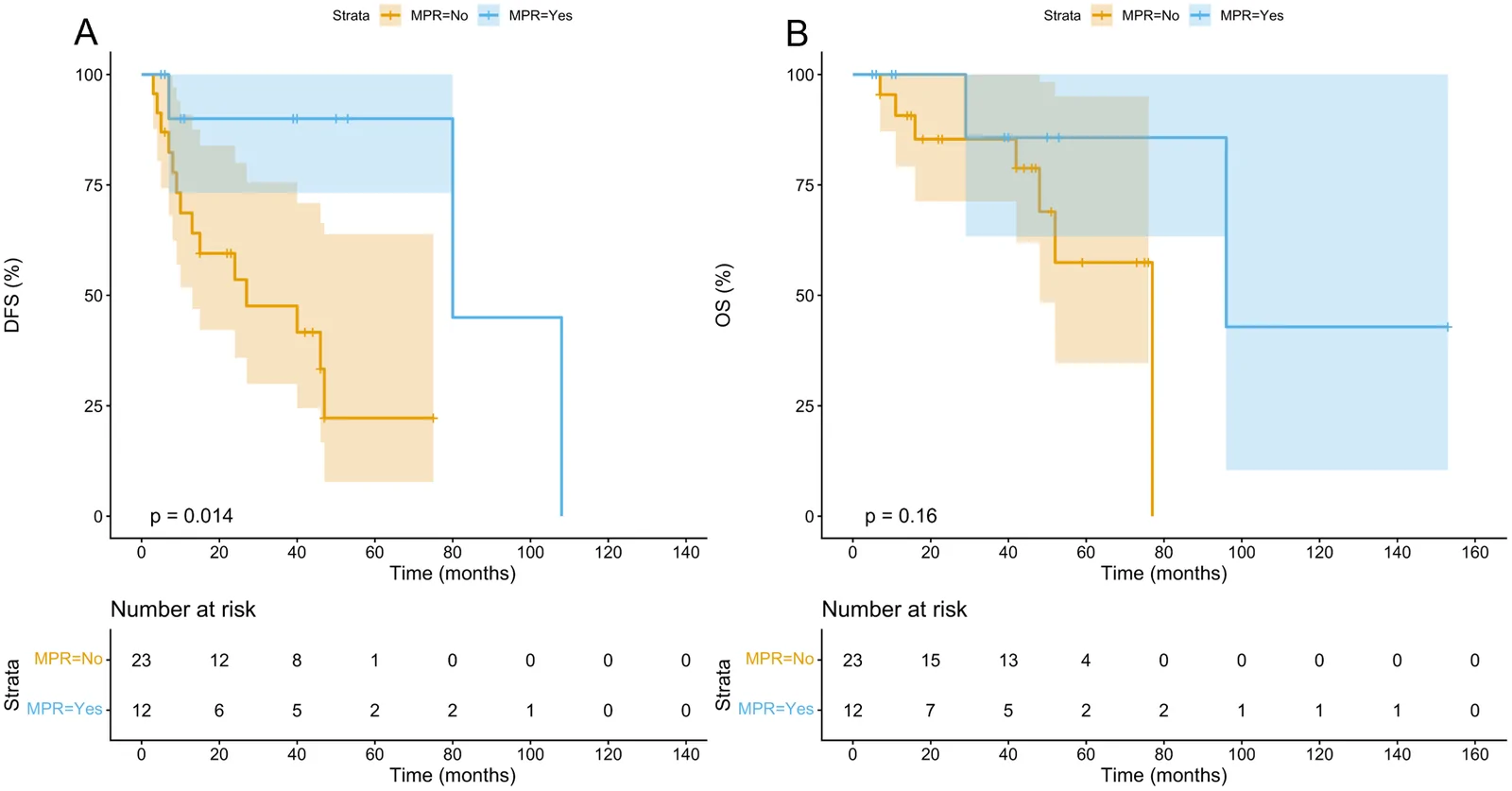
**(a)** Disease free survival according to MPR **(b)** Overall survival according to MPR.

### 3.3 Toxicity profile

Major wound complications, as defined by NCI Canada, were observed in 14 patients (37.8% 95% CI: 22.5%–55.2%) in this study. All but one of them had an extremity sarcoma. The median largest pre-treatment dimension of these patients was 11.05cm. Seven (50%) of these patients were histologic grade 3. Major wound complications occurred in 6 of 16 (37.5%) proton SFRT and 8 of 21 (38.1%) photon SFRT patients (RD -0.6%, 95% CI -32.2% to 32.0%; RR 0.98, 95% CI 0.42 to 2.21; [p =1.000]) (**Table 2**). Furthermore, there was no significant association between NCIC major wound complication and the tumor size (P = 0.452) or histologic grade (P = 0.213) among the cases. Acute grade 2–3 toxicities, graded per CTCAE5, occurred in 17 patients (45.94%); all but one were grade 2, with one patient with grade 3 radiation dermatitis. The most common were grade 2 skin-related toxicities (moist desquamation, radiation dermatitis, and hyperpigmentation) in 11 patients (29.73%), followed by grade 2 pain in 10 patients (27.0%). Other grade 2 toxicities included fatigue, nausea/vomiting, and weight loss. Acute grade 2–3 toxicity occurred in 6 of 16 (37.5%) proton SFRT versus 11 of 21 (52.4%) photon SFRT patients (RD -14.9%, 95% CI -44.9% to 19.7%; RR 0.72, 95% CI 0.32 to 1.45; [p = 0.508]). Late toxicities occurred in 3 of 16 (18.8%) proton SFRT versus 9 of 21 (42.9%) photon SFRT patients (RD -24.1%, 95% CI -50.7 to 10.1; RR 0.44, 95% CI 0.14 to 1.21; [p = 0.166]) (**Supplementary Table S4**). No between modality toxicity comparison reached statistical significance.

**Table 2:** Cross tabulation of NCIC major wound complication by radiation technique.

Variable	Proton SFRT n (%)	Photon SFRT n (%)	RD (95% CI)	RR (95% CI)	P value*
NCIC major wound complication (N = 37)					
No	10 (62.5%)	13 (61.9%)			1.000
Yes	6 (37.5%)	8 (38.1%)	-0.6% (-32.2% – 32.0%)	0.98 (0.42 to 2.21)	

*Fisher’s exact.

## 4 Discussion

This study represents the largest comparative analysis of proton versus photon spatially fractionated radiotherapy (SFRT) in soft tissue sarcomas (STS) to date. Despite the known higher proximal/surface dose deposition with protons, there was no increase in perioperative or skin toxicities compared with photon SFRT in our series. Perioperative and skin toxicity rates did not significantly differ between proton and photon SFRT when integrated into a preoperative regimen with conventional fractionated radiation and surgery. Despite underlying physical dose disparities, these results offer a promising, hypothesis-generating rationale for proton SFRT.

Prior work by Mohiuddin et al. reported early clinical outcomes of proton SFRT with limited follow-up (5.9 months) in a small mixed cohort, while Ahmed et al. described high response rates in advanced sarcomas, though only 3 of 53 patients received protons (19, 25). Our study expands upon these early reports by directly comparing toxicity and outcomes between modalities in a homogeneous curative-intent population. Sixteen patients (43.2%) in our cohort received proton GRID, making this the largest series to date comparing proton and photon SFRT.

Proton SFRT demonstrated a lower D90/D10 ratio (median 0.038 vs. 0.093), indicating higher intratumoral dose heterogeneity. While proton beams deliver increased surface and proximal tissue doses due to their physical properties (16, 17), this did not translate into higher rates of skin or wound complications. Major wound complications occurred in 37.8% of patients— with no statistically significant difference between modalities—and consistent with established benchmarks for preoperative RT, including the NCIC SR-2 trial (35% overall; 43% for lower extremity tumors), RTOG 0630 (36.6%), and HYPORT-STS (31%) (20, 26–28). The wide confidence interval inherent to a 37-patient series precludes definitive conclusions about excess toxicity. Notably, 75.7% of our cohort had extremity sarcomas with a median tumor size of 11.8 cm, making the 37.8% rate comparable to the 43% lower extremity subgroup rate from the SR-2 trial (20). These data suggest that the addition of SFRT does not increase wound morbidity beyond what is expected with standard preoperative RT (**Supplementary Table S5**).

In this cohort, a major pathological response (MPR), defined as ≥80% tumor necrosis, was observed in 12 of 35 evaluable patients (34.3%), independent of pre-treatment tumor size. Because MPR (≥80% necrosis) and the pathological complete response (0% viable tumor) reported in the RTOG trials are defined differently, these rates are not directly comparable and no superiority over historical RTOG outcomes is implied (**Supplementary Table S5**) (21, 28, 29). MPR did not differ by radiation technique in our series. Similarly, the DFS benefit noted in patients with MPR on exploratory Kaplan Meier analysis should be interpreted with caution, given the limited number of patients and events. Further work is therefore needed to determine whether the dosimetric advantages of proton SFRT translate into improved pathological or clinical outcomes over photon SFRT. Importantly, no grade 4 or 5 toxicities were observed, and late toxicity rates (32.43%) did not differ significantly between modalities. Although no toxicity comparison reached statistical significance, the confidence intervals were wide and clinically relevant differences between modalities could not be excluded. The absence of a statistically significant difference does not demonstrate equivalence or non-inferiority; establishing either would require a prespecified margin and adequate power, which this retrospective cohort lacks. These findings should therefore be regarded as hypothesis-generating.

Limitations of this study include the relative heterogeneity of presentations and additional neoadjuvant treatments with SFRT+CFRT. Thirteen patients received neoadjuvant systemic therapy and 9 patients received hyperthermia, proton SFRT was more likely done from 2020 onwards (**Supplementary Table S6**). Additional patient numbers along with extended follow up will be required to further evaluate oncological outcomes and determine whether SFRT modality or other neoadjuvant therapies improve oncological outcomes. The local failure rate observed in this cohort should be interpreted with caution given the wide confidence intervals. Definitive conclusions would require a larger cohort. The strong association between positive margins and local recurrence (P = 0.002) suggests that margin status, rather than the SFRT approach, was the primary driver of local failures. The retrospective nature of the study is an additional limitation. The radiation technique groups (proton SFRT vs photon SFRT) were imbalanced with respect to ypT4 disease, which was more common in the proton arm. Although this difference did not reach conventional statistical significance (p = 0.054), the imbalance is clinically relevant and, given the non-randomized allocation, may reflect selection bias. Because ypT stage reflects post-treatment pathological extent rather than a baseline characteristic, it should not be interpreted as a pre-treatment prognostic covariate. Despite these limitations, this series does provide initial signals there is not a difference in peri-operative complications.

Preclinical studies by Bekker and others suggest SFRT may stimulate antitumoral immune responses (30). In our cohort, concurrent neoadjuvant immunotherapy in a subset of patients was feasible and safe, reinforcing the potential synergy between SFRT and immunomodulatory agents.

In summary, this analysis provides hypothesis generating signal that proton SFRT achieves similar toxicity and oncologic outcomes compared to photon SFRT despite inherent physical dose differences. These findings support further prospective evaluation in larger multi-institutional studies.

## Data Availability

The datasets presented in this article are not readily available because due to institutional and patient privacy constraints governed by our Institutional Review Board (IRB) protocol, the data generated and analyzed for this study are not available for public access. Sharing of these data is strictly limited to the approved research team. Requests to access the datasets should be directed to jmolitoris@umm.edu.

## Supplementary material

Supplementary Figure 1 **(a)** Proton GRID plan, dose distributions, and GTV **(b)** Photon GRID plan, dose distributions, and GTV.

Supplementary Figure 2 Overall freedom from Local Recurrence.

Supplementary Figure 3 Scatter plot of Percent pathologic necrosis vs Largest pre-treatment (Radiologic) Dimension (cm).
