# Supplementary material for: Clinical outcomes and toxicity after proton- versus photon-based spatially fractionated radiotherapy for soft-tissue sarcoma: a retrospective cohort
Source: Front Oncol. 2026 Oct 28;16:1741156. doi: 10.3389/fonc.2026.1741156 (PMC13751515; doi:10.3389/fonc.2026.1741156)
Supplement: Supplementary file 1 [file DataSheet1.docx]

Table S1: Demographic and Clinicopathologic Data stratified per SFRT modality

| Variable | Photon SFRT n (%) | Proton SFRT n (%) | P Value* |
| --- | --- | --- | --- |
| Gender |  |  | 0.088 |
| Male | 15 (71.4) | 7 (43.8) |  |
| Female | 6 (28.6) | 9 (56.2) |  |
|  |  |  |  |
| Race |  |  | 0.075 |
| White | 10 (47.6) | 11 (68.8) |  |
| Black | 10 (47.6) | 2 (12.5) |  |
| Asian | 0 (0.0) | 1 (6.2) |  |
| Other | 1 (4.8) | 2 (12.5) |  |
|  |  |  |  |
| ECOG PS |  |  | 0.435 |
| 0 | 17 (81.0) | 10 (62.5) |  |
| 1 | 3 (14.2) | 5 (31.2) |  |
| 2 | 1 (4.8) | 1 (6.3) |  |
|  |  |  |  |
| Site |  |  | 0.393 |
| Extremity | 17 (81.0) | 11 (68.8) |  |
| Non extremity | 4 (19.0) | 5 (31.2) |  |
|  |  |  |  |
| Histological Grade (n=33) |  |  | 0.595 |
| G1 | 1 (5.6) | 0 (0.0) |  |
| G2 | 6 (33.3) | 5 (33.3) |  |
| G3 | 11 (61.1) | 10 (66.7) |  |
|  |  |  |  |
| Median (pre-treatment) size cm (IQR) | 11.8 (9.1-14.9) | 11.7 (10.0-15.6) | 0.818^ |
|  |  |  |  |
| T Stage** |  |  | 0.054 |
| ypT1 | 1 (4.8) | 0 (0.0) |  |
| ypT2 | 9 (42.8) | 4 (25.0) |  |
| ypT3 | 10 (47.6) | 6 (37.5) |  |
| ypT4 | 1 (4.8) | 6 (37.5) |  |

* Likelihood ratio
^ Mann-Whitney U
** ypT is a post treatment pathologic variable not a baseline characteristic

Table S2: Cross tabulation of Margin status by Local recurrence

| Variable | Margin Status |  | P Value* |
| --- | --- | --- | --- |
|  | Negative | Positive |  |
| Local recurrence (N = 37) |  |  |  |
| Yes | 3 (9.4%) | 4 (80.0%) | 0.002^ |
| No | 29 (90.6%) | 1 (20.0%) |  |

*Fisher’s exact
^Statistically significant

Table S3: Cross tabulation of MPR by radiation technique

| Variable | Photon SFRT n (%) | Proton SFRT n (%) | P Value* |
| --- | --- | --- | --- |
| MPR (N = 35) |  |  | 0.489 |
| No | 12 (60.0) | 11 (73.3) |  |
| Yes | 8 (40.0) | 4 (26.7) |  |
|  |  |  |  |
| Local Recurrence (N = 37) |  |  |  |
| No | 17 (81.0) | 13 (81.3) | 1.000 |
| Yes | 4 (19.0) | 3 (18.8) |  |

*Fisher’s exact

Table S4: Crosstabulation of Acute and Late toxicities by radiation technique

| Variable | Photon SFRT n (%) | Proton SFRT | P Value* |
| --- | --- | --- | --- |
| Acute G2/G3 toxicities (N = 37) |  |  | 0.508 |
| No | 10 (47.6) | 10 (62.5) |  |
| Yes | 11 (52.4) | 6 (37.5) |  |
|  |  |  |  |
| Late toxicities (N = 37) |  |  |  |
| No | 12 (57.1) | 13 (81.3) | 0.166 |
| Yes | 9 (42.9) | 3 (18.8) |  |

*Fisher’s exact

Table S5: Comparison with other sarcoma trials

| **Trial** | **N** | **RT Approach** | **Median Size (cm)** | **% Grade 3^d^** | **NCIC SR2 MWC Rate^a^** | **pCR/MPR Rate^e^** |
| --- | --- | --- | --- | --- | --- | --- |
| NCIC SR-2 (O'Sullivan 2002) | 94 (preop arm) | 50 Gy / 25 fx preop | Not reported^f^ | Not reported  (83% intermediate/high) | 35% (43% lower extremity)^b^ | Not reported |
| RTOG 9514 (Kraybill 2006) | 64 | 44 Gy split-course + MAID chemo | 15cm | 73.4% | Not reported (11% per local definition, not SR2 NCIC)^c^ | 27.5% |
| RTOG 0630 (Wang 2015) | 79 | 50 Gy / 25 fx, reduced IGRT margins | 10.5 cm | 48.1% | 36.6% (42% lower extremity)^b^ | 19.4% |
| HYPORT-STS (Guadagnolo 2022) | 120 | 42.75 Gy / 15 fx preop | 7.6 cm | 51% | 31% | Not reported |
| MC1973 (Ahmed 2025) | 117 (surgical) | 42.75 Gy / 15 fx preop | 6.6 cm | 57.1% | 23.1% | Not reported |
| Current series | 37 | SFRT (12–15 Gy × 1) + 50.4 Gy CFRT preop | 11.8 cm | 56.8% | 37.8% | 34.3% (MPR not pCR) |

Abbreviations: MWC, major wound complication; pCR, pathologic complete response; MPR, major pathologic response; IGRT, image-guided radiation therapy; MAID, mesna, doxorubicin, ifosfamide, dacarbazine; fx, fractions.

ᵃ MWC is defined according to the NCIC SR-2 criteria (secondary operation for wound repair, or invasive wound management, readmission for wound care, or prolonged deep packing within 120 days of surgery), as stated in each trial’s methods. Denominators: NCIC SR-2, 31/88 evaluable preoperative patients; RTOG 0630, 26/71; HYPORT-STS, 37/120; MC1973, 27/117.

ᵇ The NCIC SR-2 lower-extremity rate (30/70) is derived from O’Sullivan 2002 (Table 3) and reported in Wang 2015. RTOG 0630 lower-extremity rate: 26/62 (42%).

ᶜ RTOG 9514 did not use NCIC SR-2 criteria. 11% (7/61) had category II or III wound complications, defined as complications delaying postoperative chemotherapy or threatening tissue or limb loss.

ᵈ Grade 3 percentages are not directly comparable across trials:

- RTOG 9514 and RTOG 0630: central pathology review, 3-tier grading (Wang 2023; Wang 2015). RTOG 9514 local grading gave 80% (Kraybill 2006).
- HYPORT-STS: FNCLCC grading.
- MC1973: grading system not specified.
- NCIC SR-2: reported only as low vs intermediate/high, system not specified.
- Unknown grade: RTOG 0630, 8.9%; RTOG 9514, 7.8%; MC1973, 14.3%; HYPORT-STS, 19%.

ᵉ pCR is defined as 0% viable tumor on central pathology review. RTOG 9514: 14/51 evaluable (Kraybill 2006; Wang 2023). RTOG 0630: 14/72 evaluable (Wang 2023). The current series reports MPR (defined as 80% necrosis), which is not directly comparable to pCR.

ᶠ NCIC SR-2 did not report median tumor size; 65% of preoperative-arm tumors were ≤10 cm.

Note: Pathological response endpoints are not defined identically across studies and the rates shown should not be interpreted as a direct head-to-head comparison. In the present cohort, MPR was defined as ≥80% tumour necrosis in the resected specimen (institutional endpoint, evaluable in 35 of 37 patients; 12/35, 34.3%). In RTOG 9514 and RTOG 0630, pCR was defined as 0% viable tumour (100% necrosis/fibrosis). Because a ≥80% necrosis threshold is less stringent than complete absence of viable tumour, the MPR rate reported here is expected to be numerically higher than an RTOG-defined pCR rate and does not indicate superior pathological response or treatment efficacy relative to the RTOG regimens. The RTOG figures are provided for descriptive context only.

Table S6 Modality Stratified Descriptive Summary

| **Characteristic** | **Proton SFRT (n=16)** | **Photon SFRT (n=21)** |
| --- | --- | --- |
| Treatment period (range) | Apr 2020 to Dec 2023 | Aug 2009 to Jul 2022 |
| SFRT technique — GRID vs lattice, n (%) |  |  |
| GRID n (%) | 15 (93.75) | 21 (100) |
| Lattice n (%) | 1 (6.25) | 0 (0) |
| Neoadjuvant systemic therapy, n (%) | 2 (12.5) | 11 (52.4) |
| Hyperthermia, n (%) | 6 (37.5) | 3 (14.3) |
| ypT4 disease, n (%) | 6 (37.5) | 1 (4.8) |

b

a

Fig S1 a: Proton GRID plan, dose distributions, and GTV b: Photon GRID plan, dose distributions, and GTV (note the photon and proton plans are from different patients)

Fig S2 Overall freedom from Local Recurrence

Fig S3 Scatter plot of Percent pathologic necrosis vs Largest pre-treatment (Radiologic) Dimension (cm)
